# Clinical Perspectives on the Use of Subcutaneous and Oral Formulations of Semaglutide

**DOI:** 10.3389/fendo.2021.645507

**Published:** 2021-06-29

**Authors:** Baptist Gallwitz, Francesco Giorgino

**Affiliations:** ^1^ Department of Medicine IV - Diabetes, Endocrinology, Nephrology, Tübingen University Hospital, Tübingen, Germany; ^2^ Department of Emergency and Organ Transplantation, University of Bari Aldo Moro, Bari, Italy

**Keywords:** glucagon-like peptide-1 receptor agonist (GLP-1RA), oral, subcutaneous, semaglutide, type 2 diabetes

## Abstract

Early and effective glycemic control can prevent or delay the complications associated with type 2 diabetes (T2D). The benefits of glucagon-like peptide-1 receptor agonists (GLP-1RAs) are becoming increasingly recognized and they now feature prominently in international T2D treatment recommendations and guidelines across the disease continuum. However, despite providing effective glycemic control, weight loss, and a low risk of hypoglycemia, GLP-1RAs are currently underutilized in clinical practice. The long-acting GLP-1RA, semaglutide, is available for once-weekly injection and in a new once-daily oral formulation. Semaglutide is an advantageous choice for the treatment of T2D since it has greater efficacy in reducing glycated hemoglobin and body weight compared with other GLP-1RAs, has demonstrated benefits in reducing major adverse cardiovascular events, and has a favorable profile in special populations (e.g., patients with hepatic impairment or renal impairment). The oral formulation represents a useful option to help improve acceptance and adherence compared with injectable formulations for patients with a preference for oral therapy, and may lead to earlier and broader use of GLP-1RAs in the T2D treatment trajectory. Oral semaglutide should be taken on an empty stomach, which may influence the choice of formulation. As with most GLP-1RAs, initial dose escalation of semaglutide is required for both formulations to mitigate gastrointestinal adverse events. There are also specific dose instructions to follow with oral semaglutide to ensure sufficient gastric absorption. The evidence base surrounding the clinical use of semaglutide is being further expanded with trials investigating effects on diabetic retinopathy, cardiovascular outcomes, and on the common T2D comorbidities of obesity, chronic kidney disease, and non-alcoholic steatohepatitis. These will provide further information about whether the benefits of semaglutide extend to these other indications.

## Introduction

For patients with type 2 diabetes (T2D), early control of hyperglycemia after diagnosis is important to prevent debilitating long-term complications and to reduce diabetes-related mortality (1, 2). This is illustrated by the results from a recent registry analysis including 34,737 patients, which showed that glycated hemoglobin (HbA_1c_) levels between 7.0% and <8.0% (53 to <64 mmol/mol) for the first year after diagnosis were associated with a greater risk of future microvascular complications (hazard ratio [HR] 1.39; 95% confidence interval [CI] 1.23–1.58), macrovascular events (HR 1.29; 95% CI 1.20–1.38), and mortality (HR 1.29; 95% CI 1.10–1.51) compared with levels of <6.5% (<48 mmol/mol) (2).

Glycemic management in patients with T2D has become more individualized, and there are now several different treatment options available, with various factors influencing the most appropriate choice for individual patients. Glucagon-like peptide-1 (GLP-1) receptor agonists (GLP-1RAs) are a well-established class of glucose-lowering agents that act on multiple pathophysiological defects in T2D, providing effective glycemic control, weight loss, and a low risk of hypoglycemia, with a well‑characterized safety profile (3). In addition, as described by Smits and van Raalte in this supplement (4), certain GLP-1RAs have also been shown to reduce the risk of cardiovascular (CV) events, as well as some renal-related endpoints, in CV outcomes trials (CVOTs) (5–8).

This article will review the place of GLP-1RAs in therapy and, within this class, specifically discuss some clinical considerations around the use of the long-acting GLP-1RA, semaglutide, when given subcutaneously or *via* its new oral formulation.

## What is the place of GLP-1RAS in Therapy?

Metformin is the first-line therapy of choice for most patients with T2D; however, if patients do not achieve their individualized HbA_1c_ target after 3–6 months, another glucose-lowering medication should be added (9). In 2018, the American Diabetes Association (ADA)/European Association for the Study of Diabetes (EASD) consensus for the management of hyperglycemia in T2D presented a new decision algorithm and, as part of this, key patient characteristics should be assessed including the existence of comorbidities, such as atherosclerotic CV disease (CVD), chronic kidney disease (CKD), or heart failure (HF), which necessitate the preferential use of certain classes of glucose-lowering agents as second-line therapy (9, 10).

In patients who have established atherosclerotic CVD or evidence of high atherosclerotic CVD risk, the ADA/EASD consensus now recommends either a GLP-1RA or a sodium-glucose co-transporter-2 inhibitor (SGLT2i) (if estimated glomerular filtration rate [eGFR] is adequate) with proven efficacy to reduce the risk of CV events (9, 11). This change represents a shift in diabetes management beyond glycemic control alone and was based on CVOTs, which demonstrated that several GLP-1RAs and SGLT2is reduced the risk of major adverse CV events (MACE; CV death, nonfatal myocardial infarction, and nonfatal stroke) compared with placebo (5–8, 12, 13). A 2019 update to the ADA/EASD consensus, based on results from the REWIND CVOT with dulaglutide, suggests that a GLP-1RA or SGLT2i should also be considered in high-risk T2D patients without established CVD but with indicators of high CV risk, such as age ≥55 years with coronary, carotid, or lower-extremity artery stenosis >50%, left ventricular hypertrophy, an eGFR <60 mL/min/1.73 m^2^, or albuminuria (8, 11). Of note, beneficial outcomes observed in CVOTs do not appear to be restricted to patients with elevated HbA_1c_, and the 2019 update of the ADA/EASD consensus suggests that GLP-1RAs or SGLT2is should be considered independently of baseline HbA_1c_ or the individualized HbA_1c_ target in patients at high CV risk (11). In recent guidelines from the European Society of Cardiology on diabetes, prediabetes, and CVD, in collaboration with the EASD, a GLP-1RA or SGLT2i with proven CVD benefit is recommended as an add-on therapy to metformin and even as a first-line therapy in drug-naïve or metformin-intolerant patients with T2D and CVD or at high or very high CV risk (14).

For patients in which HF or CKD predominates, the ADA/EASD consensus recommends an SGLT2i with evidence of reducing HF and/or CKD progression, or if SGLT2is are not tolerated or contraindicated or if eGFR is less than adequate, a GLP-1RA with proven CV benefit can be added (11). If further treatment intensification is needed after second-line SGLT2i therapy, a GLP-1RA may be added (11). Recent results from a meta-analysis indicate greater reductions in HbA_1c_, body weight, and systolic blood pressure with a lower requirement of rescue therapy when a GLP-1RA was added in combination with an SGLT2i vs. SGLT2i monotherapy alone (15).

For patients without CVD, the ADA/EASD consensus advocates involving specific factors that could impact on the choice of treatment, including the need to avoid weight gain and/or hypoglycemia, in the decision cycle (9, 11). In addition, the importance of choosing treatment regimens to optimize adherence and persistence is emphasized (9). For patients without established CVD but with a compelling need to minimize weight gain or promote weight loss, either a GLP-1RA with good efficacy for weight loss or an SGLT2i is recommended (9, 11). For patients without established CVD but with a compelling need to minimize hypoglycemia, a GLP-1RA, an SGLT2i, a dipeptidyl peptidase-4 inhibitor, or a thiazolidinedione are the recommended options. A sulfonylurea or a thiazolidinedione should be considered when cost is a major issue.

### Current Underutilization

Despite being effective glucose-lowering therapies with CV and renal benefits, GLP-1RAs are often underutilized. A nationwide analysis in Denmark found that, while the use of GLP-1RAs has increased since their introduction in 2005, they still only accounted for 8% of all glucose-lowering drugs used in 2017 (16). In a survey of patients who initiated a GLP-1RA in Northern Italy over the period 2010 to 2018 (*N* = 5,408), it appeared that over time GLP-1RAs were being prescribed to patients with progressively more advanced disease, with significant increases in baseline age, diabetes duration, presence of CVD, and insulin use in patients receiving GLP-1RA therapy during the study period (17).

This apparent delay in prescribing GLP-1RAs and intensifying treatment, despite poor glycemic control in a substantial proportion of patients, was also seen in a UK survey of 113 physicians who contributed data for 1,096 patients (18). The median time from diagnosis to GLP-1RA initiation was 6.1 years and patients had HbA_1c_ values above 7.0% for a median of 13.5 months prior to switching from their last oral regimen to a GLP-1RA. In a UK physician perceptions survey completed in 2014, factors that most commonly caused hesitation when prescribing GLP-1RAs included that they were not considered first-line therapy according to guidelines, their injectable mode of administration, cost, and the potential for gastrointestinal (GI) adverse effects (19). The most common reasons reported for prescribing GLP-1RAs were weight loss, good efficacy, and low hypoglycemia risk.

## Development of GLP-1RAS and Semaglutide

Although GLP-1RAs act *via* the same overall mechanism, they vary structurally, and differ in their pharmacokinetics and clinical specifics (**Table 1**), with some degree of heterogeneity in respect to their ability to reduce HbA_1c_ and body weight, and evidence of cardiorenal protection (27, 28). The first GLP-1RAs to be developed needed to be administered subcutaneously twice daily (exenatide (20)) or once daily (lixisenatide (21) and liraglutide (22)). Subsequent developments led to the approval of longer-acting GLP-1RAs that could be administered once weekly (exenatide extended release [ER] (23), dulaglutide (24), and semaglutide (25)) to reduce the injection burden and improve convenience. Indeed, once-weekly regimens have been associated with better adherence than more frequently dosed agents (exenatide vs. liraglutide) (29), and this may lead to improved outcomes.

**Table 1:** Summary of the clinical particulars of available GLP-1RAs (20–26).

	Exenatide	Lixisenatide	Liraglutide	Exenatide ER	Dulaglutide	Semaglutide	
Route	Subcutaneous	Subcutaneous	Subcutaneous	Subcutaneous	Subcutaneous	Subcutaneous	Oral
Frequency	Twice daily	Once daily	Once daily	Once weekly	Once weekly	Once weekly	Once daily
Timing of administration	Within 60 mins of the morning and evening meal	Within 60 mins of any meal (preferably the same meal each day)	Any time (independent of meals) but preferably the same time each day	Any time of day, with or without meals	Any time of day, with or without meals	Any time of day, with or without meals	On an empty stomach 30 mins before eating, drinking, or taking other oral medications
Dosage regimens	Starting: 5 μg	Starting: 10 μg	Starting: 0.6 mg	No up-titration	No up-titration	Starting: 0.25 mg	Starting: 3 mg
	Maintenance: 10 μg	Maintenance: 20 μg	Maintenance: 1.2 mg & 1.8 mg	Maintenance: 2 mg	Maintenance: 0.75 mg for monotherapy or 1.5 mg as add-on (a starting dose of 0.75 mg may be used in vulnerable patients)	Maintenance: 0.5 mg & 1.0 mg	Maintenance: 7 mg & 14 mg
Are dose adjustments needed in special populations?							
Elderly	Exercise caution and proceed conservatively with escalation to 10 μg if >70 years	None needed based on age	None needed based on age	None needed based on age	None needed based on age	None needed based on age	None needed based on age
Renal impairment							
Mild	None	None	None	None	None	None	None
Moderate	Proceed conservatively with escalation to 10 μg	None	None	None	None	None	None
Severe	Not recommended	Not recommended	None	Not recommended	None	None	None
ESRD	Not recommended	Not recommended	Not recommended	Not recommended	Not recommended	Not recommended	Not recommended
Hepatic impairment	None	None	Not recommended with severe impairment	None	None	Exercise caution with severe impairment	Exercise caution with severe impairment

ER, extended release; ESRD, end-stage renal disease; GLP-1RA, glucagon-like peptide-1 receptor agonist.

Semaglutide has 94% sequence homology with native GLP-1, with three key structural differences that prolong its half-life to approximately one week, without compromising GLP-1 receptor binding (25, 30). In the SUSTAIN program, subcutaneous semaglutide consistently demonstrated superior and sustained glycemic control and weight loss compared with comparators across the T2D disease continuum (31). As reviewed by Meier in this supplement (32), in head-to-head trials with other long-acting GLP-1RAs, subcutaneous semaglutide 1.0 mg produced superior HbA_1c_ and weight reductions compared with exenatide ER 2.0 mg (estimated treatment difference [ETD] –0.62% and 3.78 kg; both *p* < 0.0001, respectively) (33) and with dulaglutide 1.5 mg (ETD –0.41% and 3.55 kg; both *p* < 0.0001, respectively) (34). Since the approval of once-weekly subcutaneous semaglutide in 2017/2018, further information is being gathered through an ongoing series of prospective, noninterventional real-world studies across 10 different countries, which aim to determine its efficacy, safety, and treatment satisfaction in patients in local clinical practice over approximately 30 weeks of treatment (35–43).

It is known that some patients prefer oral over injectable medications (44, 45), and lower treatment adherence has been reported with more frequent administration or when patients perceive the treatment as difficult or inconvenient (45, 46). Oral medication may also help to overcome the clinical inertia seen in the frequent reluctance to initiate injectable medicines. For this reason, an oral formulation of semaglutide was developed and was approved for the treatment of adults with T2D by the U.S. Food and Drug Administration in September 2019 and by the European Medicines Agency in April 2020. In Europe, subcutaneous semaglutide and oral semaglutide are indicated as adjuncts to diet/exercise either as monotherapy, when metformin is considered inappropriate due to intolerance or contraindications, or in combination with other glucose-lowering medication(s), for patients who do not have sufficient glycemic control (25, 26). As the first oral formulation of a GLP-1RA, oral semaglutide represents a useful option to help improve acceptance and adherence compared with injectable formulations in those patients with a preference for oral therapy, and may contribute to the reversal of current underutilization, potentially leading to earlier initiation of GLP-1RAs in the T2D disease continuum.

## Dosing Considerations With Subcutaneous and Oral Semaglutide

### Dose Escalation

As a class, GLP-1RAs have a well-defined safety profile. The most commonly reported adverse events (AEs) are GI-related effects, including nausea, diarrhea, and vomiting, which are generally mild-to-moderate in severity and transient in nature (47). In general, GI AEs are most frequent shortly after treatment initiation and therefore slow up-titration of the dose is recommended for most GLP-1RAs (**Table 1**). For subcutaneous semaglutide, the starting dose is 0.25 mg once weekly, and after 4 weeks, the dose should be increased to 0.5 mg once weekly (25). After at least 4 weeks on a dose of 0.5 mg once weekly, the dose can be increased to 1 mg once weekly to further improve glycemic control. For oral semaglutide, patients should start treatment with the 3 mg dose once daily for 1 month, then increase to 7 mg once daily (26). After at least 1 month on a dose of 7 mg once daily, the dose can be increased to a maintenance dose of 14 mg once daily if needed to further improve glycemic control. When starting semaglutide, patients should be reassured that GI AEs do not affect the majority of patients and are likely to be only mild-to-moderate in severity and transient (25, 26). To help minimize any nausea, patients could be advised to eat smaller meals and stop when they feel full, and to avoid meals with a high fat content (48–50).

### Dosing Instructions

Subcutaneous semaglutide can be dosed at any time on the day of the weekly injection, with or without meals (25). For oral semaglutide, the presence of food in the stomach impairs absorption (51, 52). Patients are advised to swallow the oral semaglutide tablet on an empty stomach, with a sip of water (up to half a glass of water equivalent to 120 mL), and to wait at least 30 minutes before eating, drinking, or taking other oral medications (26). This may be problematic for some patients, and may influence their preferred choice of formulation.

**Figure 1:**
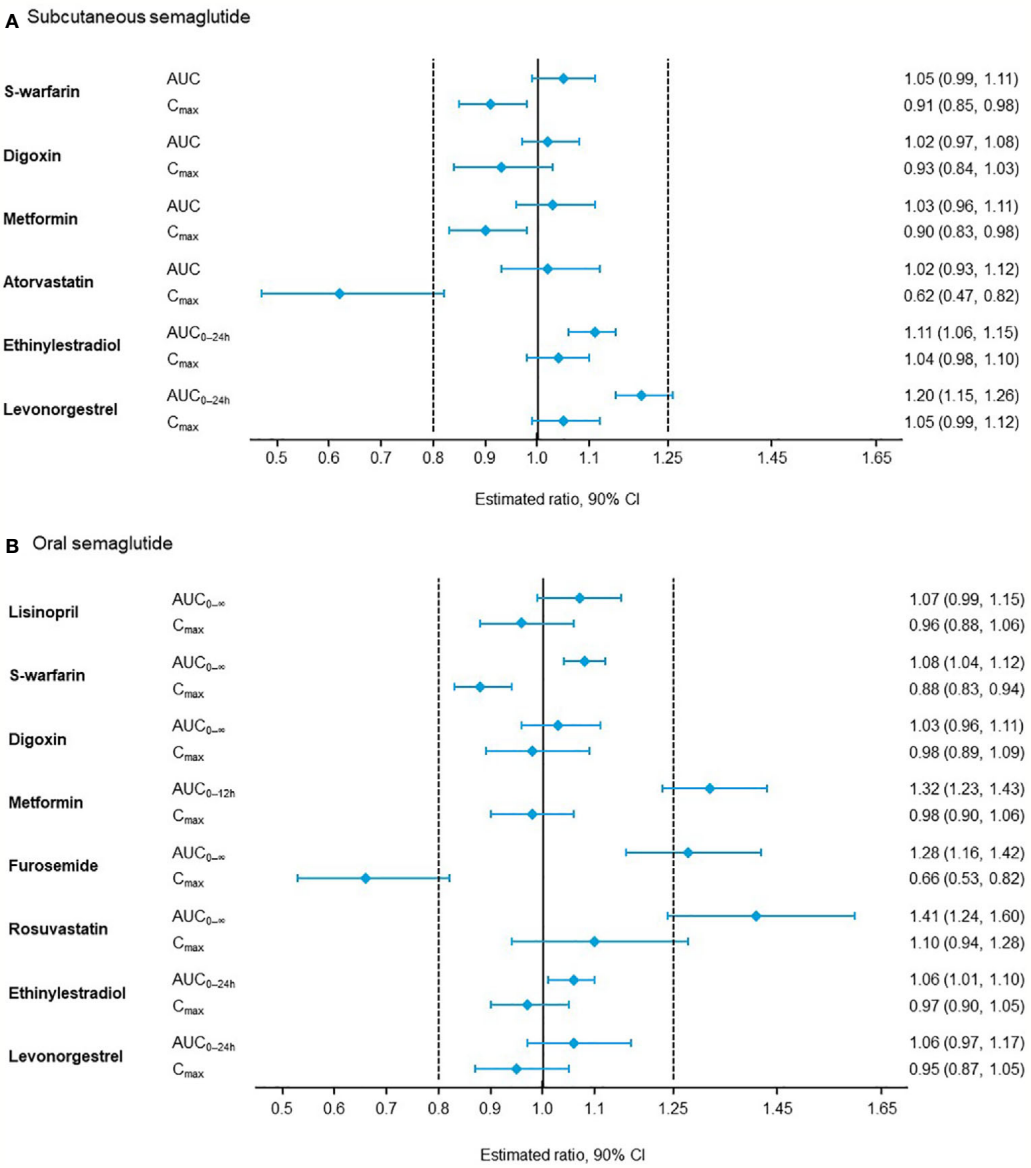
Effect of **(A)** subcutaneous semaglutide and **(B)** oral semaglutide on the pharmacokinetics of co-administered drugs (53–57). AUC, area under the curve; CI, confidence interval; C_max_, maximum concentration.

In pharmacokinetic studies, subcutaneous or oral semaglutide did not have clinically relevant effects on the exposure of other widely used medications, such as warfarin, metformin, digoxin, atorvastatin/rosuvastatin (53–55), or the combined oral contraceptive, ethinylestradiol/levonorgestrel (**Figure 1**) (56, 57). In addition, oral semaglutide did not have clinically relevant effects on the exposure of lisinopril or furosemide (53, 54). When tested with omeprazole, which increases gastric pH, no clinically relevant interactions were observed on the exposure of oral semaglutide (58).

In a drug–drug interaction study, levothyroxine exposure was increased by 33% when co-administered with oral semaglutide 14 mg, which may be due to delayed gastric emptying and increased levothyroxine absorption (59). Monitoring of thyroid parameters should therefore be considered when treating patients with oral semaglutide at the same time as levothyroxine (26). When co-administering other oral medications, it is important to adhere to the administration instructions for oral semaglutide, and consider increased monitoring for medications that have a narrow therapeutic index or that require clinical monitoring (60).

In population pharmacokinetic and exposure−response analyses, the exposure range following oral semaglutide was wider than for subcutaneous dosing but with a considerable overlap between oral semaglutide 7 and 14 mg and subcutaneous semaglutide 0.5 and 1.0 mg (61). The effect of switching between oral and subcutaneous semaglutide cannot easily be predicted because of the high pharmacokinetic inter-individual variability of oral semaglutide; however, exposure after 14 mg oral semaglutide once daily appears comparable with 0.5 mg subcutaneous semaglutide once weekly (26). It is recommended that patients switching from once-weekly subcutaneous semaglutide at a dose of 0.5 mg can be transitioned onto oral semaglutide at a dose of 7 or 14 mg once daily, up to 7 days after their last injection of subcutaneous semaglutide; however, there is no equivalent oral dose for those switching from subcutaneous semaglutide 1 mg (60).

## Semaglutide in Renal Impairment

CKD is a common complication of T2D and a major cause of morbidity and mortality (62). The exendin-4-based GLP-1RAs, exenatide (immediate-release and ER) and lixisenatide are partially renally eliminated and are not recommended in patients with severe renal impairment (eGFR <30 mL/min/1.73 m^2^) (20, 21, 23) (**Table 1**). Furthermore, dose escalation of immediate-release exenatide should proceed conservatively in patients with moderate renal impairment (eGFR 30–50 mL/min/1.73 m^2^) (21). Results from pharmacokinetic studies have established that dose adjustments are not necessary when semaglutide (subcutaneous or oral) is used in patients with different levels of renal impairment (25, 26, 63, 64). Like all other GLP-1RAs, semaglutide is not recommended in patients with end-stage renal disease (ESRD) (eGFR <15 mL/min/1.73 m^2^) (25, 26).

To provide further data on the use of semaglutide in patients with renal dysfunction, the PIONEER 5 trial evaluated the efficacy and safety of once-daily oral semaglutide 14 mg vs. placebo in 324 patients with T2D and moderate renal impairment (eGFR 30–59 mL/min/1.73 m^2^) (65). Superior and significant reductions in HbA_1c_ and body weight were observed with oral semaglutide vs. placebo over 26 weeks, and renal function was unchanged throughout the study in both treatment groups. Patients with CKD were also included in the SUSTAIN 6 and PIONEER 6 CVOTs (6, 66). Indeed, in SUSTAIN 6, the CKD-related endpoint of new or worsening nephropathy was found to occur in significantly fewer patients in the subcutaneous semaglutide group compared with the placebo group (3.8% vs. 6.1%; HR 0.64; 95% CI 0.46–0.88; *p* = 0.005) (6).

GLP-1RAs may exert beneficial actions on the kidneys through reductions in blood glucose, blood pressure, and weight, as well as *via* possible direct cardio-nephroprotective mechanisms, such as improved endothelial dysfunction, reduced oxidative stress, and reduced inflammation (62). The phase III FLOW trial (NCT03819153) is ongoing to determine the effect of once-weekly subcutaneous semaglutide 1.0 mg vs. placebo on the progression of renal impairment in over 3,000 patients with T2D and CKD (eGFR 50–75 mL/min/1.73 m^2^ and urinary albumin-to-creatinine ratio [UACR] >300–<5,000 mg/g or eGFR 25–50 mL/min/1.73 m^2^ and UACR >100–<5,000 mg/g) (67). The primary endpoint is the time to the first occurrence of a composite primary outcome event, defined as persistent eGFR decline of ≥50% from trial start, reaching ESRD, death from kidney disease, or death from CVD for up to 5 years.

## Semaglutide in Hepatic Impairment

There is a complex interplay between T2D and liver disease, particularly non-alcoholic fatty liver disease (NAFLD) and non-alcoholic steatohepatitis (NASH), which are common in patients with T2D (68). The mechanisms responsible for the link between NAFLD and T2D are not completely understood but could include genetic factors, insulin resistance, dysfunctional adipose tissue, chronic hyperglycemia, altered gut microbiome, and changes in hepatokines, among others (68, 69).

GLP-1RAs appear to be well-tolerated in patients with hepatic impairment, and dose adjustments are not necessary (**Table 1**) (20–26). Consistent with this, pharmacokinetic studies have established no apparent effect of hepatic impairment on the exposure of semaglutide when each formulation was tested (25, 26, 70, 71).

Novel therapies are in demand for the treatment of NAFLD, and early studies suggested that GLP-1RAs may reduce liver inflammation and fibrosis (72). Potential mechanisms for the GLP-1RAs’ benefit in the context of NAFLD include: reduced body weight and body fat through central regulation of satiety; reduced hepatic, skeletal muscle, and adipose tissue insulin resistance due to decrease in body weight; modified intestinal lipoprotein metabolism; and amelioration of dysfunctional adipose tissue and enhancement of insulin release (72, 73). The safety and efficacy of liraglutide 1.8 mg once daily for 48 weeks were tested in a phase II trial in 52 patients with NASH, in which this drug was found to be well-tolerated (74). Furthermore, there was evidence of histological resolution in the end-of-treatment biopsy in 39% of patients in the liraglutide group compared with only 9% in the placebo group.

A phase II trial recently evaluated the effects of once-daily subcutaneous semaglutide (0.1 mg, 0.2 mg, and 0.4 mg) vs. placebo in 320 patients with NASH (75). Treatment with semaglutide 0.4 mg resulted in a significantly higher percentage of patients achieving the primary endpoint of NASH resolution and no worsening of fibrosis than placebo after 72 weeks (59% vs. 17%; *p* < 0.001).

Given the lack of hepatic GLP-1 receptor expression, the potential mechanism of action by which semaglutide results in NASH resolution may be mediated *via* weight loss. However, semaglutide is also associated with improvements in insulin resistance, hepatic lipotoxicity, and hepatic inflammation. In pre-clinical models, improvements in inflammation with liraglutide were shown to be independent of weight reduction, as was prevention of initiation of fibrosis (76). Thus, it appears unlikely that improvements in NASH with GLP-1 receptor agonists are solely mediated *via* weight reduction.

## Semaglutide in Obesity

Compared with other GLP-1RAs, the capability for weight loss appears to be higher with semaglutide, and the ADA/EASD consensus provides the following ranking for weight-loss efficacy: subcutaneous semaglutide > liraglutide > dulaglutide > exenatide > lixisenatide (9). The mechanisms responsible for weight loss have been investigated for both subcutaneous and oral semaglutide (77, 78). In 30 patients with obesity, *ad libitum* energy intake was substantially lower with once-weekly subcutaneous semaglutide (dose escalated to 1.0 mg) vs. placebo for 12 weeks, and this was associated with reduced appetite and food cravings, better control of eating, and lower preference for fatty, energy-dense food (77). Subcutaneous semaglutide induced a 5.0 kg reduction in mean body weight after 12 weeks, which was found to be derived predominantly from body fat mass reduction, assessed by air displacement plethysmography. Consistent results have been observed with once-daily oral semaglutide (dose escalated to 14 mg) vs. placebo in a similar study in 15 patients with T2D (78).

A phase II dose-finding trial evaluated the efficacy and safety of once-daily subcutaneous semaglutide in promoting weight loss (79). In total, 957 patients with obesity (body mass index [BMI] ≥30 kg/m^2^) but without T2D were randomized to once-daily subcutaneous semaglutide (dose escalated to 0.05 mg, 0.1 mg, 0.2 mg, 0.3 mg, or 0.4 mg), once-daily subcutaneous liraglutide (dose escalated to 3.0 mg), or placebo, in combination with dietary and physical activity counseling, with the primary endpoint of percentage weight loss at week 52. Estimated mean weight change was –2.3% for the placebo group and ranged from –6.0% with subcutaneous semaglutide 0.05 mg to –13.8% with subcutaneous semaglutide 0.4 mg after 52 weeks (all *p* ≤ 0.001). Furthermore, mean body weight reductions with semaglutide at a dose of 0.2 mg or higher were significantly greater than with liraglutide (–7.8%).

These findings paved the way for the phase III STEP (Semaglutide Treatment Effect in People with obesity) program, which is currently investigating body weight changes following treatment with once-weekly 2.4 mg subcutaneous semaglutide (80). This global clinical program has enrolled approximately 5,000 adults with overweight or obesity. The main eligibility criteria for weight in the STEP 1, 3, 4, and 5 trials were BMI ≥30 kg/m^2^ or BMI ≥27 kg/m^2^ with at least one weight-related comorbidity (hypertension, dyslipidemia, obstructive sleep apnea, or CVD), while patients in STEP 2 had to have a BMI ≥27 kg/m^2^ and T2D. The primary endpoint of STEP 1–5 is the change from baseline to end of treatment in body weight; the proportion of patients achieving a body weight reduction of ≥5% is a co-primary endpoint in STEP 1–3 and 5. In the completed STEP trials, semaglutide 2.4 mg as an adjunct to lifestyle intervention led to mean body weight losses of ~15–17% over 68 weeks in patients without T2D (STEP 1, 3 and 4), with a smaller mean weight loss of 9.6% seen in patients with T2D over the same period (STEP 2). At week 68, 86–89% of patients without T2D achieved ≥5% body weight loss (STEP 1, 3 and 4), with 69% of patients with T2D achieving this threshold (STEP 2). Across all studies, semaglutide 2.4 mg also demonstrated benefits beyond weight loss on cardiometabolic parameters and patient-reported outcomes (81–84).

In addition to the STEP program, the effect of semaglutide treatment on CV outcomes is being assessed in adults aged ≥45 years with overweight or obesity. The SELECT phase III trial (NCT03574597) is investigating whether once-weekly subcutaneous semaglutide (up to 2.4 mg) can reduce MACE vs. placebo in approximately 17,500 people with overweight or obesity and established CVD with a follow-up of approximately 5 years (85).

## Additional Large-Scale Ongoing Studies With Semaglutide

Following the phase III programs for subcutaneous and oral semaglutide, additional questions remain that are being investigated in ongoing studies. In the CVOT, SUSTAIN 6, subcutaneous semaglutide was associated with a higher risk of diabetic retinopathy complications than placebo after 2.1 years (6). Most events occurred early in the trial, and this has been suggested to be attributable to the magnitude and rapidity of the HbA_1c_ reduction in patients with pre-existing diabetic retinopathy (86). Patients with proliferative retinopathy or maculopathy resulting in active treatment were excluded from the PIONEER 6 CVOT, in which no apparent imbalance was observed between oral semaglutide and placebo in the AE reporting of diabetic retinopathy over 16 months (66). The long-term FOCUS phase III trial (NCT03811561) is currently ongoing to specifically investigate the effects of subcutaneous semaglutide on diabetic retinopathy complications (87). Approximately 1,500 patients with T2D and Early Treatment Diabetic Retinopathy Study (ETDRS) level of 10–75 in both eyes and no ocular or intraocular treatment for diabetic retinopathy or diabetic macular edema in the 6 months prior to screening will receive once-weekly subcutaneous semaglutide 1.0 mg or placebo for up to 5 years, with the primary endpoint of progression of 3 steps or more in ETDRS level.

Subcutaneous semaglutide significantly reduced the rate of MACE vs. placebo in a *post-hoc* non-prespecified analysis of SUSTAIN 6, but it is unknown whether oral semaglutide can also reduce CV events (6). In PIONEER 6, oral semaglutide significantly reduced the rate of MACE and decreased all-cause mortality vs. placebo. However, while oral semaglutide was demonstrated to be noninferior to placebo in PIONEER 6, the trial was not powered to assess any potential CV benefit (66).

SOUL (NCT03914326) is an ongoing CVOT evaluating the effects of once-daily oral semaglutide (up to 14 mg) vs. placebo in 9,642 patients with T2D and CVD, cerebrovascular disease, symptomatic peripheral artery disease, or CKD (88). The primary endpoint is time to the first occurrence of MACE, with a follow-up of approximately 5 years. Secondary endpoints will explore the effects of oral semaglutide on other CV endpoints and assess any improvements in additional diabetic complications, including CKD and limb ischemia.

## Conclusions

The benefits of GLP-1RAs are becoming increasingly recognized in international T2D recommendations and, along with other agents targeted at T2D pathophysiology, such as SGLT2is, their initiation early in the disease trajectory is advocated. The higher efficacy of semaglutide in reducing HbA_1c_ and body weight compared with other GLP-1RAs and favorable clinical characteristics make semaglutide, either subcutaneous or oral, an advantageous choice for T2D treatment. Oral semaglutide provides an additional treatment option for patients and physicians who may be reluctant to initiate or intensify therapy by injection, and this may also help to increase earlier GLP-1RA utilization.

Where unanswered questions remain about the impact of semaglutide on outcomes, ongoing trials are underway to provide additional clarity. Effects on diabetic nephropathy and retinopathy are being assessed for subcutaneous semaglutide, and whether there are any positive CV benefits of oral semaglutide will also be determined. The management of comorbidities that are increasingly common in patients with T2D, such as obesity and liver disease, need to be better addressed; in this respect, ongoing trials will provide further information about whether the benefits of semaglutide extend to these other indications.

## Author Contributions

All authors contributed to the article and approved the submitted version.

## Funding

This article was supported by Novo Nordisk, who was provided with the opportunity to perform a medical accuracy review.

## Conflict of Interest

FG has received research support from Eli Lilly, Lifescan, and Takeda, and has provided advisory services to AstraZeneca, Boehringer Ingelheim, Eli Lilly, Lifescan, Merck Sharp & Dohme, Novo Nordisk, Roche Diabetes Care, and Sanofi. BG has provided advisory services to AstraZeneca, Bayer, Boehringer Ingelheim, Eli Lilly, Merck Sharp & Dohme, and Novo Nordisk, and has received lecture honoraria from Bristol Myers Squibb and the above-mentioned companies.

The authors declare that this article received funding from Novo Nordisk. The funder had the following involvement in the article: medical writing support.
